# A new technique to resolve Nasal Rhinosporidiosis: A case report with review of literature

**DOI:** 10.1016/j.ijscr.2022.106807

**Published:** 2022-02-04

**Authors:** Vidya G. Doddawad, Ranbir Singh, Shivananda S

**Affiliations:** a Department of Oral Pathology and Microbiology, JSS Dental College and Hospital, A constituent college of JSS Academy of Higher Education & Research, Mysore 570022, India; b North Bazar, Kaviraj gali, P.O. Andal, Pashim Burdawan, West Bengal 713321, India; c Department of Oral and Maxillofacial Surgery, JSS Dental College and Hospital, A constituent college of JSS Academy of Higher Education & Research, Mysore 570022, India

**Keywords:** Rhinosporidiosis, Chronic granulomatous disease, Mucosal polyp, *Rhinosporidium seeberi*, Surgical excision, Dapsone

## Abstract

**Introduction and importance:**

Rhinosporidiosis is a chronic, localized granulomatous infectious disease caused by *Rhinosporidium seeberi* that predominantly affects the mucosal membranes of the nose and nasopharynx, conjunctiva, and urethra. *Rhinosporidium seeberi* is a eukaryotic pathogen that spreads in certain geographical areas, particularly in tropical and subtropical areas, through aquatic exposure.

**Case presentation:**

We present the case of a young man who had been suffering from a right nasal mass for four months, and whose diagnosis was confirmed after surgical excision and histopathological examination, which revealed distinct pathognomonic findings. Laser-assisted endoscopic excision, in combination with Dapsone, is recommended as a more effective treatment to prevent a recurrence.

**Clinical discussion:**

For clinicians, it has been advised to obtain a detailed case history of exposure in patients diagnosed with Rhinosporidiosis. Rhinosporidiosis can be diagnosed with a simple examination of H&E-stained histopathological sections. Because chemotherapy has not been proven to be effective, Laser-assisted endoscopic excision, in combination with Dapsone is the recommended treatment for Rhinosporidiosis.

**Conclusion:**

One of the differential diagnoses for Rhinosporidiosis in the nasal cavity is masses or abnormal growths without bleeding, which should be kept in mind by clinicians and pathologists.

## Highlights

• Laser-assisted endoscopic excision, Dapsone, is a more effective treatment for Rhinosporidiosis to prevent a recurrence.

## 1 Introduction and importance

Rhinosporidiosis is a chronic granulomatous disease caused by *Rhinosporidium seeberi* which was misleading as fungi, parasites and bacteria by taxonomic classification. However, based on genetic sequencing and the nature of aquatics, it was later identified as an aquatic eukaryote [1]. In tropical and subtropical countries, a large number of cases have been reported, with the disease spreading through stagnant water or contaminated soil [2].

Rhinosporidiosis is characterized by polypoid tumor-like masses in mucosal sites such as the nasal, oral, and genital, etc. These lesions are friable and pendulous. Nasopharyngeal Rhinosporidiosis cases present with rhinorrhea, epistaxis, and, eventually, obstruction. The clinical presentation and symptoms, as well as histological findings, are used to make a diagnosis in these cases [3], [4]. We present a case of Rhinosporidiosis, focusing on the clinical presentation, histological features, diagnosis, and management for recurrence prevention.

This work has been reported in line with the SCARE 2020 criteria [3].

## 2 Case presentation

**Fig. 1:**
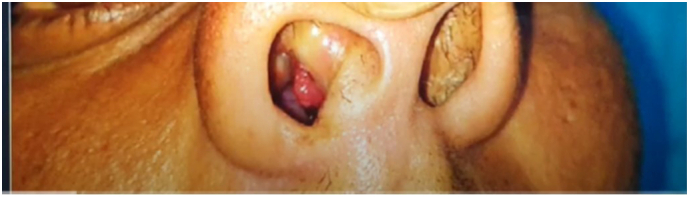
Clinical photograph showing reddish, polypoidal mass protruding from the right nasal cavity.

A 35-year-old man presented to our hospital with a mass on the right side of his nose that had been present for four months. The patient worked as a farmer and had no chronic medical conditions. After further investigation, he admitted to taking a bath in a pond. On clinical examination, a polypoid, strawberry-coloured, pedunculated hard mass measuring 1 × 2 cm was found in the right side nasal cavity, with no bleeding on probing and no history of epistaxis (Fig. 1).

Patient was not under any medication. However, we could not access other family members for physical examination to identify possible source of infection. There were no any other oral habits like smoking, tobacco chewing.

Blood tests were unremarkable; a routine CBC examination revealed a 10% increase in eosinophils, and other biochemical tests (LFT, RFT) were within normal limits.

**Fig. 2:**
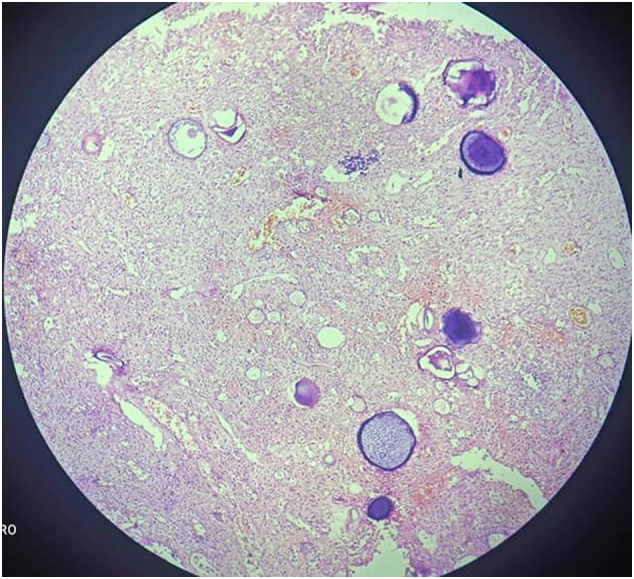
HE staining section showing connective stroma shows mature sporangia with spores and immature sporangia, chronic inflammatory cell infiltrate, and blood vessels (100× magnification).

**Fig. 3:**
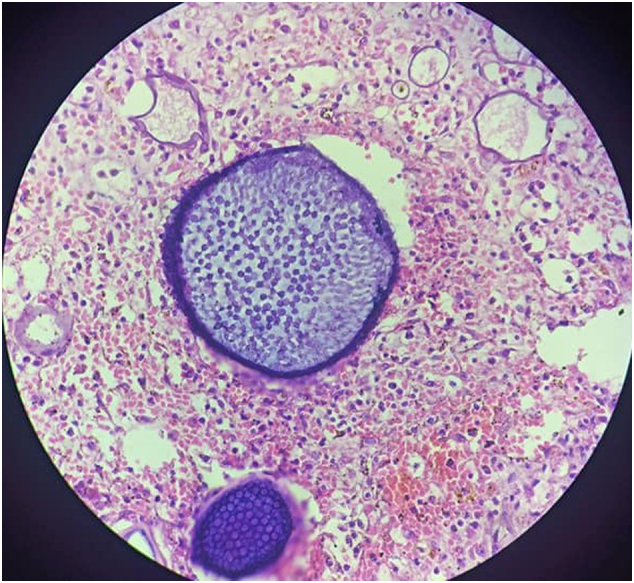
HE staining section showing mature sporangia with central mature endospores and peripheral small immature endospores (400× magnification).

Laser-assisted endoscopic excision was used to completely remove the mass and performed by oral and maxillofacial surgeon. The excised specimen was sent for histopathological examination to confirm the diagnosis. Hyperplastic squamous epithelium and oedematous fibro-connective tissue with many globular cysts, fibroblasts, and inflammatory cells such as neutrophils, lymphocytes, and plasma cells are found on histopathological examination. Each cyst contained a large number of thick-walled pathognomonic sporangia, which contained a large number of endospores at various stages of development. We could diagnose Nasal Rhinosporidiosis based on all of these clinical and histologic findings (Fig. 2, Fig. 3).

A patient was reviewed for a one-year follow-up and noticed to have a good prognosis without recurrence or any complication. Advised post-operative instructions to a patient not to take bath in a pond/lake. The patient's clinical profile, intra-operative findings, and typical histopathological findings were all used to diagnosis the patient.

## 3 Discussion

Rhinosporidiosis is a rare and misunderstood infectious disease that was discovered after a certain year based on its etiology. *Rhinosporidium seeberi*, an aquatic eukaryote and a hydrophilic organism that infects humans and animals, cause the disease. It was named after R. Seeber, who first described this lesion in Argentina and identified a causative agent as a possible etiology, based on the clinical presentation. Aquatic animals such as fish and reptiles are the most common hosts for this organism, while humans, domestic mammals, and birds are rare hosts [3], [4].

Guillemo Seeber reported this pathogen as a parasite that appears as nasal polyps in animals and humans in the 1890s, and it was named Coccidium seeberia after the protozoal subdivision Coccidia. Later in the early 1900s, Ashworth discovered that the organism's life cycle differed from that of a parasite, so he studied and named as ‘*Rhinosporidium seeberi*’ under the fungus category [3], [5]. Many microbiologists and pathologists now consider it to be a fungus, but its taxonomy is still up for debate. Ragan et al. identified the 18S rRNA gene in *Rhinosporidium seeberi* by phylogenetic analysis, as it is in the DRIP clade (acronym derived from Dermocystidium, Rosette agent, Ichthyophonus and Psorospermum). Many aspects of *Rhinosporidium seeberi* infection, such as taxonomy, morphology, ontogenesis, and epidemiology, are still debated. However, most microbiologists assumed it as a fungus due to its ability to stain with fungal stains like GMS and PAS. Researchers discovered that this microbe lives in a water reservoir as well as contaminated soil using fluorescent in-situ hybridization techniques [6].

The disease is most common in tropical and subtropical areas, with the majority of infections occurring in Asia's major endemic regions, such as south India and Sri Lanka, but infections have also been reported in the Americas, Europe, and Africa [6], [7]. Currently, our patient is from a Rhinosporidiosis-endemic region in southeast India, which serves as an excellent example [6].

Stagnant lakes and ponds, as well as contaminated soil/sand, are the modes of transmission, which have been linked to their occupations as swimmers and agriculturists. Other modes of transmission have been reported, including autoinfection from endospore spillage during trauma/surgery, hematogenous dissemination, lymphatic spread, and sexual transmission [6], [8]. To establish the epidemiological link, we were able to obtain a history of frequent activities involving water exposure, such as bathing in a pond, during our assessment. This could be a sign that the host has predisposing factors. *Rhinosporidium seeberi* enters the mucosa through the traumatized epithelium because the pathogen is thought to require wet surfaces for attachment and proliferation [6]. It's unclear whether the infection is caused by a genetic predisposition [6], [9].

Rhinosporidiosis can affect any mucosal site in the human body, causing friable, polypoid tumor-like masses to appear. The majority of affected patients are adults between the ages of 20 and 40, with a male-to-female ratio of about 2.5:1 [4]. However, some authors claim that the male-to-female ratio is 4:1 in patients between the ages of 20 and 35 [10]. The reason for this is that males are more active in their occupations, outdoor activities, and animal contact than females. The epidemiology of rhinosporidiosis is still unknown because it is necessary to determine whether rhinosporidiosis is acquired in specific communities or if there are any unknown factors. The nasal cavity/nasopharynx is the most affected site, followed by ocular, cutaneous, throat, ear, genital, respiratory mucosa [6], bone, larynx, and oesophagus [3], [6]. This lesion is found on the nose in 53% to 58% of cases, the nasopharynx in 12% to 17% of cases, the ocular region in 10% of cases, and other sites in 30% of cases [3]. Our case involved a 35-year-old farmer who had a history of bathing in a pond, which was consistent with Nasal Rhinosporidiosis.

Nasal Rhinosporidiosis usually develops gradually, and the vascular friable polyps with projections that bleed easily give it a “strawberry” appearance. Foreign body sensation, rhinorrhoea, nasal obstruction, and epistaxis are all symptoms that patients may experience [4]. All of the clinical signs and symptoms were the same, with the exception that there was no bleeding in our case.

Various studies have suggested that nonspecific immune reactivity in the host, blood group, and HLA types may be important in the pathogenesis of Rhinosporidium seeberi in establishing an initial infection focus [6]. Rhinosporidiosis could be caused by a decrease in anti-rhinosporidial cell-mediated immunity as a result of the switch from Th-1 to Th-2.

Tuberculosis, angiofibromas, nasopharyngeal carcinomas, and papilloma are the differential diagnoses for Rhinosporidiosis based on clinical and radiographic findings [11]. All of these, however, were ruled out due to histological findings.

The pathogen can be identified using biopsy specimens, which explains the histopathologic characteristics. The stroma, which surrounds the sporangia and sporangiospores, contains fibrous connective tissue as well as inflammatory cells such as lymphocytes, macrophages, and neutrophils. Sporangia are spherical structures with thick walls that contain smaller “daughter cells” called “sporangiospores” [7], [12]. The current case had all of the above characteristics, so it was diagnosed as Rhinosporidiosis.

In the submucosa of the affected site, the infectious agent forms round and thick-walled sporangia and endospores that range in size from 10 to 20 μm and 50 to 1000 μm, respectively [3]. Fungal stains such as Gomori methenamine silver (GMS), Gridley's, mucicarmine, and periodic acid-Schiff (PAS), as well as routine stain like hematoxylin and eosin (H&E) staining, can also be used to detect it [10]. Under 10% KOH or Papanicolaou stain (PAP), the FNAC examination can be performed. Advanced molecular techniques such as PCR or 16s-RNA can also be used to confirm the diagnosis [6].

The *Rhinosporidium seeberi* is differs from another microorganism, *Coccidioides immitis* in two aspects [3].

1. The structure of *Coccidioides immitis* is large, thick-walled, spherical structures containing endospores which are having a diameter of 20–80 μm but *Rhinosporidium seeberi* are 50–1000 μm in dimension.
2. Mucicarmine does not stain Coccidioides immitis, but *Rhinosporidium seeberi* does.

Adjuvant antifungal drug therapies such as griseofluvin and amphotericin B, trimethoprim-sulphadiazine, and sodium stibogluconate were previously tried with varying degrees of success. Despite the fact that all of these drugs were endosporestatic rather than endosporicidal, but *Rhinosporidium seeberi* is resistant to antimicrobial drugs. Only a few cases with a high recurrence rate were reported. The line of treatment for Rhinosporidiosis is wide surgical excision along with cauterization of the base of the lesion and chemotherapy like Dapsone was prescribed to prevent a recurrence. The mode of action of Dapsone is to prevent the maturation of the sporangia and promote fibrosis in the stroma [13]. The failure of chemotherapy due to the impenetrability of the organism's cell wall and the difficulty of culturing the organism, making susceptibility testing an in vitro method [10].

Currently, Laser-assisted endoscopic excision, in combination with Dapsone, may be used to treat nasal/nasopharyngeal Rhinosporidiosis and prevent recurrences. To solve the problem in this case, the same treatment method was used. Many authors have noted that while no spontaneous disease regression has been observed in animals, it is highly doubtful in humans [6].

Despite all of these treatments, recurrence rates remain high, ranging from 10% to 70%, due to sporangia spillage and seeding during removal, which invade adjacent normal tissues, or incomplete surgical excision removal [14], [15]. Since the one-year follow-up in our case, the lesion has not recurred.

The lesion can spread to the nasopharynx, oropharynx, and maxillary antrum if the patient neglected to treat it or the clinician fails to identify it. Nasal septal perforation can cause significant morbidity due to continuous haemorrhage. This could be the complication of Rhinosporidiosis [6].

## 4 Conclusion

One of the differential diagnoses for Rhinosporidiosis in the nasal cavity is masses or abnormal growths without bleeding, which should be kept in mind by clinicians and pathologists. The evaluation of clinical findings and history, as well as the characteristic histological findings, are critical steps in arriving at a final diagnosis. For clinicians, it has been advised to obtain a detailed case history of exposure in patients diagnosed with Rhinosporidiosis. Rhinosporidiosis can be diagnosed with a simple examination of H&E-stained histopathological sections. Because chemotherapy has not been proven to be effective, Laser-assisted endoscopic excision, in combination with Dapsone is the recommended treatment for Rhinosporidiosis.

## Consent

Written informed consent was obtained from the patient for publication of this case report and accompanying images.

## Ethical approval

Ethical standards were reviewed and approved by the Department of oral pathology and microbiology.

## Funding

This research did not receive any specific grant from funding agencies in the public, commercial, or not-for-profit sectors.

## Guarantor

Vidya G Doddawad accepts full responsibility for the work and the conduct of the study, had access to the data, and controlled the decision to publish.

## Research registration number

N/A.

## CRediT authorship contribution statement

VGD, SS and RC conceptualized and prepared the manuscript. SS performed the surgery. VGD,RS prepared and reported the histology slides. All authors have read and approved the final manuscript.

## Declaration of competing interest

The authors declare they have no competing interests.
